# Di-μ_2_-acetato-1:2κ^2^ *O*:*O*′;2:3κ^2^ *O*:*O*′-bis­{μ_2_-4,4′-dichloro-2,2′-[2,2-dimethyl­propane-1,3-diylbis(nitrilo­methanylyl­idene)]diphenolato}-1:2κ^6^ *O*,*N*,*N*′,*O*′:*O*,*O*′;2:3κ^6^ *O*,*O*′:*O*,*N*,*N*′,*O*′-tricopper(II)

**DOI:** 10.1107/S1600536812044315

**Published:** 2012-10-31

**Authors:** Koji Kubono, Keita Tani, Kunihiko Yokoi

**Affiliations:** a Division of Natural Sciences, Osaka Kyoiku University, Kashiwara, Osaka 582-8582, Japan

## Abstract

The title compound, [Cu_3_(C_19_H_18_Cl_2_N_2_O_2_)_2_(CH_3_CO_2_)_2_], is a linear homo-trinuclear Cu^II^ complex. The central Cu^II^ atom is located on a centre of inversion and has a distorted octa­hedral coordination environment formed by six O atoms from two tetra­dentate Schiff base ligands and two bridging acetate ligands. The coordination geometry of the terminal Cu^II^ atom is square-pyramidal with a tetra­dentate ligand in the basal plane. The apical site is occupied by one O atom from an acetate ligand. The acetate-bridged Cu⋯Cu distance is 3.0910 (5) Å. An intra­molecular C—H⋯O hydrogen bond forms an *S*(6) ring motif. The crystal of the trinuclear complex is stabilized by C—H⋯O hydrogen bonds.

## Related literature

For the supra­molecular chemistry of related complexes, see: Chen *et al.* (2010 ▶); von Richthofen *et al.* (2009 ▶); Gianneschi *et al.* (2003 ▶). For related structures, see: Atakol *et al.* (1999 ▶); Feng *et al.* (2007 ▶); Ray *et al.* (2009 ▶); Yang *et al.* (2004 ▶). For background to this work, see: Fukuhara *et al.* (1990 ▶); Kargar *et al.* (2012 ▶); Kubono *et al.* (2009 ▶, 2010 ▶). For hydrogen-bond motifs, see: Bernstein *et al.* (1995 ▶). For analysis of ring conformations, see: Cremer & Pople (1975 ▶).
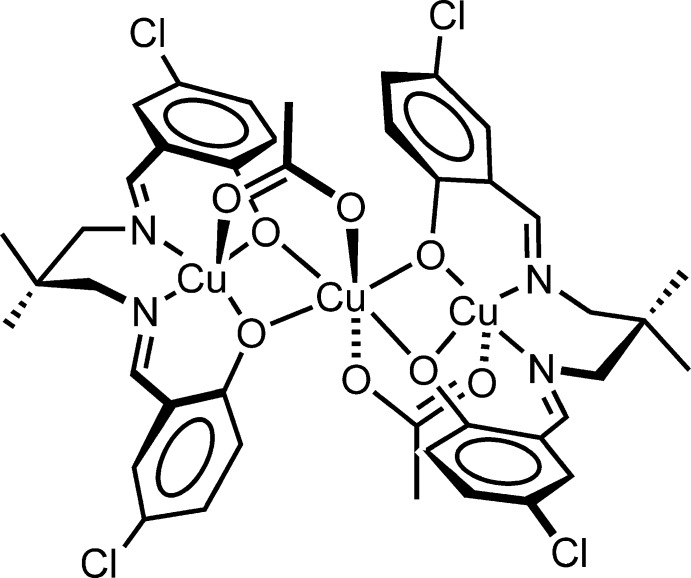


## Experimental

### 

#### Crystal data

[Cu_3_(C_19_H_18_Cl_2_N_2_O_2_)_2_(C_2_H_3_O_2_)_2_]
*M* *_r_* = 1063.25
Orthorhombic, 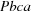

*a* = 19.0732 (18) Å
*b* = 11.6191 (11) Å
*c* = 19.693 (3) Å
*V* = 4364.2 (9) Å^3^
*Z* = 4
Mo *K*α radiation
μ = 1.75 mm^−1^
*T* = 298 K
0.23 × 0.20 × 0.16 mm

#### Data collection

Rigaku AFC7R diffractometer
Absorption correction: ψ scan (North *et al.*, 1968 ▶) *T*_min_ = 0.675, *T*_max_ = 0.756
7325 measured reflections
5006 independent reflections
2796 reflections with *F* ^2^ > 2.0σ(*F* ^2^)
*R*_int_ = 0.024
3 standard reflections every 150 reflections intensity decay: 0.5%

#### Refinement

*R*[*F* ^2^ > 2σ(*F* ^2^)] = 0.035
*wR*(*F* ^2^) = 0.105
*S* = 1.00
5006 reflections
280 parameters
H-atom parameters constrained
Δρ_max_ = 0.39 e Å^−3^
Δρ_min_ = −0.45 e Å^−3^

### 

Data collection: *PROCESS-AUTO* (Rigaku, 2006 ▶); cell refinement: *PROCESS-AUTO*; data reduction: *CrystalStructure* (Rigaku, 2010 ▶); program(s) used to solve structure: *SIR92* (Altomare *et al.*, 1993 ▶); program(s) used to refine structure: *SHELXL97* (Sheldrick, 2008 ▶); molecular graphics: *PLATON* (Spek, 2009 ▶); software used to prepare material for publication: *CrystalStructure*.

## Supplementary Material

Crystal structure: contains datablock(s) global, I. DOI: 10.1107/S1600536812044315/mw2092sup1.cif

Structure factors: contains datablock(s) I. DOI: 10.1107/S1600536812044315/mw2092Isup2.hkl

Additional supplementary materials:  crystallographic information; 3D view; checkCIF report

## Figures and Tables

**Table 1:** Hydrogen-bond geometry (Å, °)

*D*—H⋯*A*	*D*—H	H⋯*A*	*D*⋯*A*	*D*—H⋯*A*
C2—H2⋯O4^i^	0.93	2.58	3.115 (4)	117
C15—H15⋯O3^ii^	0.93	2.59	3.289 (4)	133

Symmetry codes: (i) 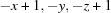; (ii) 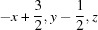.

**Fig. 1.**
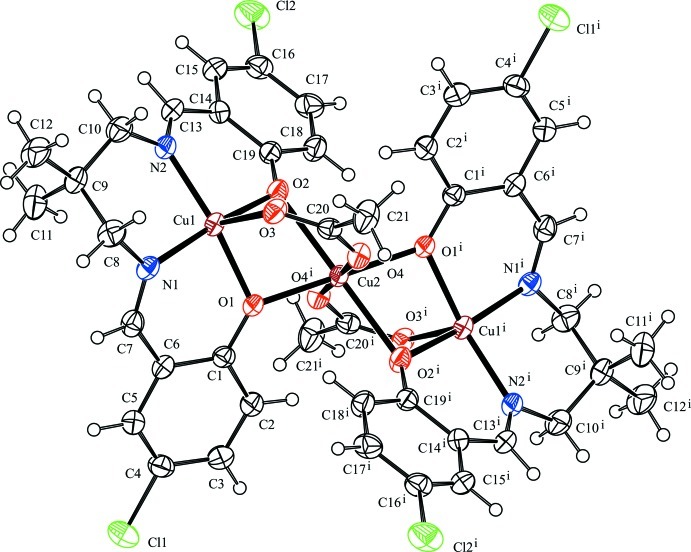
The molecule of the title complex showing the atom-labelling scheme. Displacement ellipsoids are drawn at the 40% probability level and H atoms are represented by circles of arbitrary size. [symmetry code: (i) -x + 1, -y, -z + 1.]

**Fig. 2.**
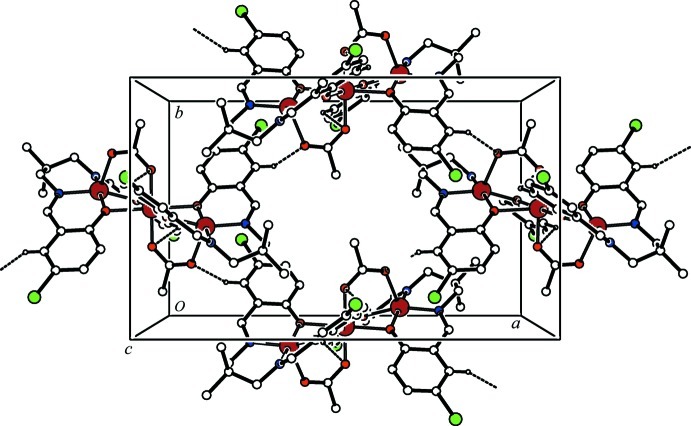
Packing diagram of the title complex, viewed down the c axis. The intramolecular and intermolecular C—H···O hydrogen bonds are shown as dashed lines.

## supplementary crystallographic information

### Comment

Supramolecular complexes, formed by hydrogen bonds or coordination linkages have received much attention, because of their interesting and functional properties such as molecular recognition, magnetism and catalysis (Chen *et al.*, 2010; von Richthofen *et al.*, 2009; Gianneschi *et al.*, 2003). We have previously studied the structures of supramolecular Cu^II^ complexes with planar tetradentate piperazine ligands containing fluoro or chloro groups (Kubono *et al.*, 2010; Kubono *et al.*, 2009). These Cu^II^ complexes form either a dimer, or a dinuclear structure through C—H···F, or C—H···Cl hydrogen bonds. Complexes with the tetradentate Schiff base ligand, bis(salicylidene)propane-1,3-diamine can form triuclear complexes with coordinating anions or solvents to generate supramolecular architectures (Atakol *et al.*, 1999; Fukuhara *et al.*, 1990; Ray *et al.*, 2009). However no structures of trinuclear complexes with bis-halogenosalicylidene and anionic ligands have been reported. We have attempted to assemble such a species from the mononuclear Cu^II^ complex {4,4'-dichloro-2,2'-[2,2-dimethylpropane-1,3- diylbis(nitrilomethanylylidene)]diphenolato}copper(II) (Kargar *et al.*, 2012) and copper(II) acetate as the building blocks. Herein, the structure of the title trinuclear complex is reported.

The central Cu^II^ atom is located on a centre of inversion and has a distorted octahedral coordination environment formed by four O atoms from two tetradentate Schiff base ligands in the equatorial plane and an O atom from each of the two bridging acetate ligands in the axial positions. The coordination geometry of the terminal Cu^II^ atom is square-pyramidal with the basal plane comprised of two phenolate O and two imine N atoms from a tetradentate ligand. The apical site is occupied by one O atom from a bridging acetate ligand. The terminal Cu^II^ atom is located 0.2370 (4) Å from the mean basal plane (N1/N2/O1/O2). The six-membered Cu1/N1/C8/C9/C10/N2 ring adopts a chair conformation with puckering parameters (Cremer & Pople, 1975): Q = 0.549 (4) Å, θ = 16.4 (3)° and φ = 141.7 (13)°. Bond lengths and angles involving Cu^II^ are comparable to those observed in related structures (Atakol *et al.*, 1999). The dihedral angle between the benzene rings (C1–C6 and C14–C19) is 68.73 (12)°. The acetate-bridged Cu···Cu distance is 3.0910 (5) Å, similar to those of related linear homo-trinuclear Cu^II^ complexes (Atakol *et al.*, 1999; Feng *et al.*, 2007; Yang *et al.*, 2004). There is an intramolecular C2—H2···O4^i^ hydrogen bond [symmetry code: (i) -*x* + 1, -*y*, -*z* + 1; Table 1], forming a *S*(6) ring motif (Bernstein *et al.*, 1995). The molecular conformationof the trinuclear complex is stabilized by the intramolecular hydrogen bonds. In the crystal, the trinuclear complex molecules are linked through intermolecular C—H···O hydrogen bonds into a two-dimensional supramolecular network, parallel to the *ab* plane (Table 1 and Fig. 2).

### Experimental

The ligand (0.40 mmol) was dissolved in 20 mL of hot methanol. Then 20 mL of a methanol solution of copper acetate monohydrate (0.60 mmol) were added to this solution.The mixture was stirred for 20 min at 340 K. After a few days at room temperature, green crystals of title complex were obtained. Yield 52%. Analysis calculated for C_42_H_42_Cl_4_Cu_3_N_4_O_8_: C 47.44, H 3.98, N 5.27%; found: C 47.48, H 3.92, N 5.21%.

### Refinement

All H atoms bound to carbon were placed in idealized positions and refined using a riding model, with C—H = 0.93–0.97 Å and *U*_iso_(H) = 1.2*U*_eq_(C).

### Crystal data

**Table:**

[Cu_3_(C_19_H_18_Cl_2_N_2_O_2_)_2_(C_2_H_3_O_2_)_2_]	*F*(000) = 2164.00
*M**_r_* = 1063.25	*D*_x_ = 1.618 Mg m^−^^3^
Orthorhombic, *P**b**c**a*	Mo *K*α radiation, λ = 0.71069 Å
Hall symbol: -P 2ac 2ab	Cell parameters from 25 reflections
*a* = 19.0732 (18) Å	θ = 15.0–17.4°
*b* = 11.6191 (11) Å	µ = 1.75 mm^−^^1^
*c* = 19.693 (3) Å	*T* = 298 K
*V* = 4364.2 (9) Å^3^	Prismatic, green
*Z* = 4	0.23 × 0.20 × 0.16 mm

### Data collection

**Table:**

Rigaku AFC7R diffractometer	*R*_int_ = 0.024
ω–2θ scans	θ_max_ = 27.5°
Absorption correction: ψ scan (North *et al.*, 1968)	*h* = −13→24
*T*_min_ = 0.675, *T*_max_ = 0.756	*k* = −8→15
7325 measured reflections	*l* = −25→0
5006 independent reflections	3 standard reflections every 150 reflections
2796 reflections with *F*^2^ > 2.0σ(*F*^2^)	intensity decay: 0.5%

### Refinement

**Table:**

Refinement on *F*^2^	Secondary atom site location: difference Fourier map
*R*[*F*^2^ > 2σ(*F*^2^)] = 0.035	Hydrogen site location: inferred from neighbouring sites
*wR*(*F*^2^) = 0.105	H-atom parameters constrained
*S* = 1.00	*w* = 1/[σ^2^(*F*_o_^2^) + (0.0428*P*)^2^ + 0.7391*P*] where *P* = (*F*_o_^2^ + 2*F*_c_^2^)/3
5006 reflections	(Δ/σ)_max_ = 0.001
280 parameters	Δρ_max_ = 0.39 e Å^−^^3^
0 restraints	Δρ_min_ = −0.45 e Å^−^^3^
Primary atom site location: structure-invariant direct methods	

### Special details

**Table:**

Geometry. All e.s.d.'s (except the e.s.d. in the dihedral angle between two l.s. planes) are estimated using the full covariance matrix. The cell e.s.d.'s are taken into account individually in the estimation of e.s.d.'s in distances, angles and torsion angles; correlations between e.s.d.'s in cell parameters are only used when they are defined by crystal symmetry. An approximate (isotropic) treatment of cell e.s.d.'s is used for estimating e.s.d.'s involving l.s. planes.
Refinement. Refinement was performed using all reflections. The weighted *R*-factor (*wR*) and goodness of fit (*S*) are based on *F*^2^. *R*-factor (gt) are based on *F*. The threshold expression of *F*^2^ > 2.0 σ(*F*^2^) is used only for calculating *R*-factor (gt).

### Fractional atomic coordinates and isotropic or equivalent isotropic displacement parameters (Å^2^)

**Table:**

	*x*	*y*	*z*	*U*_iso_*/*U*_eq_	
Cu1	0.64313 (2)	0.07637 (3)	0.44178 (2)	0.03103 (12)	
Cu2	0.5000	0.0000	0.5000	0.02727 (14)	
Cl1	0.47496 (6)	−0.12235 (10)	0.13509 (5)	0.0602 (3)	
Cl2	0.77615 (6)	−0.36381 (9)	0.66639 (5)	0.0635 (3)	
O1	0.54772 (11)	0.0350 (2)	0.40871 (11)	0.0336 (5)	
O2	0.61636 (11)	−0.01062 (19)	0.52089 (11)	0.0359 (6)	
O3	0.60366 (11)	0.23406 (18)	0.48839 (11)	0.0363 (6)	
O4	0.49941 (11)	0.16268 (18)	0.52142 (11)	0.0341 (5)	
N1	0.66389 (14)	0.1401 (2)	0.35159 (14)	0.0326 (6)	
N2	0.74426 (14)	0.0639 (2)	0.46700 (14)	0.0349 (7)	
C1	0.53159 (17)	0.0020 (3)	0.34682 (16)	0.0302 (7)	
C2	0.47113 (18)	−0.0639 (3)	0.33434 (18)	0.0384 (8)	
H2	0.4416	−0.0830	0.3702	0.046*	
C3	0.45508 (19)	−0.1003 (3)	0.27000 (18)	0.0418 (9)	
H3	0.4152	−0.1447	0.2628	0.050*	
C4	0.49741 (19)	−0.0718 (3)	0.21590 (18)	0.0405 (9)	
C5	0.55477 (18)	−0.0033 (3)	0.22513 (18)	0.0385 (8)	
H5	0.5816	0.0188	0.1879	0.046*	
C6	0.57347 (17)	0.0342 (3)	0.29035 (17)	0.0320 (8)	
C7	0.63314 (18)	0.1099 (3)	0.29713 (19)	0.0374 (8)	
H7	0.6511	0.1401	0.2570	0.045*	
C8	0.72103 (18)	0.2234 (3)	0.34661 (19)	0.0433 (9)	
H8A	0.7110	0.2877	0.3765	0.052*	
H8B	0.7229	0.2527	0.3005	0.052*	
C9	0.79266 (17)	0.1737 (3)	0.36505 (18)	0.0390 (9)	
C10	0.79631 (19)	0.1469 (3)	0.44084 (18)	0.0465 (10)	
H10A	0.8428	0.1175	0.4509	0.056*	
H10B	0.7908	0.2185	0.4656	0.056*	
C11	0.8086 (2)	0.0676 (4)	0.3228 (2)	0.0584 (11)	
H11A	0.7764	0.0072	0.3345	0.088*	
H11B	0.8557	0.0426	0.3316	0.088*	
H11C	0.8038	0.0859	0.2755	0.088*	
C12	0.8473 (2)	0.2683 (4)	0.3510 (2)	0.0592 (12)	
H12A	0.8928	0.2418	0.3646	0.089*	
H12B	0.8353	0.3362	0.3763	0.089*	
H12C	0.8477	0.2859	0.3034	0.089*	
C13	0.76749 (18)	−0.0137 (3)	0.50710 (17)	0.0407 (9)	
H13	0.8160	−0.0181	0.5115	0.049*	
C14	0.72733 (18)	−0.0952 (3)	0.54648 (16)	0.0345 (8)	
C15	0.76437 (19)	−0.1790 (3)	0.58283 (18)	0.0426 (9)	
H15	0.8130	−0.1824	0.5795	0.051*	
C16	0.72974 (19)	−0.2558 (3)	0.62314 (18)	0.0416 (9)	
C17	0.65761 (19)	−0.2510 (3)	0.63013 (18)	0.0434 (9)	
H17	0.6346	−0.3027	0.6585	0.052*	
C18	0.62008 (19)	−0.1695 (3)	0.59500 (17)	0.0408 (9)	
H18	0.5715	−0.1677	0.5993	0.049*	
C19	0.65340 (17)	−0.0887 (3)	0.55256 (16)	0.0312 (7)	
C20	0.54487 (18)	0.2416 (3)	0.51585 (16)	0.0312 (7)	
C21	0.5241 (2)	0.3555 (3)	0.5462 (2)	0.0532 (11)	
H21A	0.5584	0.4126	0.5345	0.080*	
H21B	0.5214	0.3484	0.5947	0.080*	
H21C	0.4792	0.3781	0.5287	0.080*	

### Atomic displacement parameters (Å^2^)

**Table:**

	*U*^11^	*U*^22^	*U*^33^	*U*^12^	*U*^13^	*U*^23^
Cu1	0.0229 (2)	0.0343 (2)	0.0359 (2)	−0.00107 (18)	0.00405 (18)	0.00024 (19)
Cu2	0.0223 (3)	0.0305 (3)	0.0290 (3)	−0.0010 (2)	0.0029 (2)	−0.0011 (2)
Cl1	0.0663 (7)	0.0744 (7)	0.0398 (5)	−0.0041 (6)	−0.0070 (5)	−0.0176 (5)
Cl2	0.0759 (8)	0.0619 (7)	0.0526 (6)	0.0295 (6)	−0.0149 (6)	0.0059 (5)
O1	0.0232 (12)	0.0450 (13)	0.0324 (13)	−0.0047 (11)	0.0016 (10)	−0.0044 (11)
O2	0.0274 (12)	0.0403 (14)	0.0400 (13)	0.0061 (11)	0.0050 (10)	0.0114 (12)
O3	0.0290 (12)	0.0341 (13)	0.0459 (15)	−0.0015 (11)	0.0017 (11)	−0.0057 (11)
O4	0.0318 (13)	0.0287 (12)	0.0419 (14)	−0.0008 (11)	0.0038 (11)	−0.0014 (10)
N1	0.0263 (15)	0.0311 (15)	0.0404 (17)	−0.0023 (12)	0.0009 (13)	0.0044 (13)
N2	0.0267 (15)	0.0431 (18)	0.0348 (15)	−0.0058 (13)	0.0037 (13)	−0.0025 (14)
C1	0.0291 (18)	0.0295 (18)	0.0320 (18)	0.0061 (15)	−0.0024 (15)	0.0003 (15)
C2	0.0329 (19)	0.045 (2)	0.0372 (19)	−0.0049 (16)	0.0018 (16)	−0.0017 (17)
C3	0.036 (2)	0.046 (2)	0.044 (2)	−0.0065 (18)	−0.0038 (18)	−0.0029 (18)
C4	0.042 (2)	0.046 (2)	0.034 (2)	0.0053 (19)	−0.0068 (16)	−0.0066 (17)
C5	0.034 (2)	0.046 (2)	0.0355 (19)	0.0010 (18)	0.0019 (16)	0.0012 (17)
C6	0.0263 (18)	0.0307 (18)	0.0390 (19)	0.0015 (14)	0.0011 (15)	0.0007 (15)
C7	0.035 (2)	0.0363 (19)	0.041 (2)	0.0015 (16)	0.0056 (16)	0.0098 (17)
C8	0.041 (2)	0.036 (2)	0.053 (2)	−0.0105 (17)	−0.0012 (18)	0.0059 (18)
C9	0.029 (2)	0.042 (2)	0.046 (2)	−0.0053 (16)	0.0060 (17)	0.0026 (17)
C10	0.035 (2)	0.056 (2)	0.048 (2)	−0.0185 (18)	−0.0001 (18)	0.0040 (19)
C11	0.048 (3)	0.062 (3)	0.065 (3)	0.000 (2)	0.020 (2)	−0.009 (2)
C12	0.042 (2)	0.068 (3)	0.067 (3)	−0.024 (2)	0.002 (2)	0.018 (2)
C13	0.0235 (18)	0.059 (3)	0.039 (2)	−0.0007 (17)	0.0005 (15)	−0.0009 (19)
C14	0.0304 (19)	0.043 (2)	0.0300 (18)	0.0035 (16)	0.0013 (14)	−0.0050 (16)
C15	0.031 (2)	0.056 (2)	0.040 (2)	0.0092 (18)	−0.0029 (16)	−0.0001 (19)
C16	0.045 (2)	0.042 (2)	0.037 (2)	0.0148 (19)	−0.0076 (17)	−0.0029 (17)
C17	0.050 (2)	0.043 (2)	0.038 (2)	0.0009 (19)	−0.0065 (18)	0.0115 (17)
C18	0.032 (2)	0.047 (2)	0.043 (2)	0.0013 (17)	0.0041 (17)	0.0103 (18)
C19	0.0289 (18)	0.0321 (18)	0.0325 (18)	−0.0001 (15)	0.0003 (14)	−0.0026 (15)
C20	0.0343 (18)	0.0285 (18)	0.0309 (19)	0.0034 (15)	−0.0048 (15)	0.0007 (14)
C21	0.046 (2)	0.039 (2)	0.075 (3)	0.0012 (19)	0.014 (2)	−0.019 (2)

### Geometric parameters (Å, º)

**Table:**

Cu1—O2	1.926 (2)	C7—H7	0.9300
Cu1—N1	1.965 (3)	C8—C9	1.527 (5)
Cu1—O1	1.992 (2)	C8—H8A	0.9700
Cu1—N2	1.997 (3)	C8—H8B	0.9700
Cu1—O3	2.183 (2)	C9—C11	1.518 (5)
Cu2—O4^i^	1.937 (2)	C9—C10	1.526 (5)
Cu2—O4	1.937 (2)	C9—C12	1.540 (5)
Cu2—O1	2.056 (2)	C10—H10A	0.9700
Cu2—O1^i^	2.056 (2)	C10—H10B	0.9700
Cu2—O2^i^	2.260 (2)	C11—H11A	0.9600
Cu2—O2	2.260 (2)	C11—H11B	0.9600
Cl1—C4	1.750 (4)	C11—H11C	0.9600
Cl2—C16	1.756 (4)	C12—H12A	0.9600
O1—C1	1.314 (4)	C12—H12B	0.9600
O2—C19	1.308 (4)	C12—H12C	0.9600
O3—C20	1.248 (4)	C13—C14	1.444 (5)
O4—C20	1.267 (4)	C13—H13	0.9300
N1—C7	1.272 (4)	C14—C15	1.400 (5)
N1—C8	1.461 (4)	C14—C19	1.417 (4)
N2—C13	1.277 (4)	C15—C16	1.364 (5)
N2—C10	1.477 (4)	C15—H15	0.9300
C1—C2	1.406 (5)	C16—C17	1.384 (5)
C1—C6	1.419 (4)	C17—C18	1.374 (4)
C2—C3	1.371 (5)	C17—H17	0.9300
C2—H2	0.9300	C18—C19	1.408 (4)
C3—C4	1.377 (5)	C18—H18	0.9300
C3—H3	0.9300	C20—C21	1.504 (5)
C4—C5	1.365 (5)	C21—H21A	0.9600
C5—C6	1.402 (4)	C21—H21B	0.9600
C5—H5	0.9300	C21—H21C	0.9600
C6—C7	1.445 (5)		
			
O2—Cu1—N1	169.27 (11)	N1—C8—C9	113.6 (3)
O2—Cu1—O1	84.00 (9)	N1—C8—H8A	108.8
N1—Cu1—O1	88.83 (10)	C9—C8—H8A	108.8
O2—Cu1—N2	90.96 (11)	N1—C8—H8B	108.8
N1—Cu1—N2	93.31 (11)	C9—C8—H8B	108.8
O1—Cu1—N2	161.12 (11)	H8A—C8—H8B	107.7
O2—Cu1—O3	90.51 (9)	C11—C9—C10	111.2 (3)
N1—Cu1—O3	97.66 (10)	C11—C9—C8	110.8 (3)
O1—Cu1—O3	91.44 (9)	C10—C9—C8	110.5 (3)
N2—Cu1—O3	106.83 (10)	C11—C9—C12	110.2 (3)
O4^i^—Cu2—O4	180.00 (13)	C10—C9—C12	106.9 (3)
O4^i^—Cu2—O1	89.99 (9)	C8—C9—C12	107.0 (3)
O4—Cu2—O1	90.01 (9)	N2—C10—C9	116.3 (3)
O4^i^—Cu2—O1^i^	90.01 (9)	N2—C10—H10A	108.2
O4—Cu2—O1^i^	89.99 (9)	C9—C10—H10A	108.2
O1—Cu2—O1^i^	180.00 (10)	N2—C10—H10B	108.2
O4^i^—Cu2—O2^i^	91.11 (8)	C9—C10—H10B	108.2
O4—Cu2—O2^i^	88.89 (8)	H10A—C10—H10B	107.4
O1—Cu2—O2^i^	105.35 (8)	C9—C11—H11A	109.5
O1^i^—Cu2—O2^i^	74.65 (8)	C9—C11—H11B	109.5
O4^i^—Cu2—O2	88.89 (8)	H11A—C11—H11B	109.5
O4—Cu2—O2	91.11 (8)	C9—C11—H11C	109.5
O1—Cu2—O2	74.65 (8)	H11A—C11—H11C	109.5
O1^i^—Cu2—O2	105.35 (8)	H11B—C11—H11C	109.5
O2^i^—Cu2—O2	180.0	C9—C12—H12A	109.5
C1—O1—Cu1	126.0 (2)	C9—C12—H12B	109.5
C1—O1—Cu2	130.5 (2)	H12A—C12—H12B	109.5
Cu1—O1—Cu2	99.58 (9)	C9—C12—H12C	109.5
C19—O2—Cu1	127.3 (2)	H12A—C12—H12C	109.5
C19—O2—Cu2	130.9 (2)	H12B—C12—H12C	109.5
Cu1—O2—Cu2	94.85 (9)	N2—C13—C14	127.6 (3)
C20—O3—Cu1	123.5 (2)	N2—C13—H13	116.2
C20—O4—Cu2	133.1 (2)	C14—C13—H13	116.2
C7—N1—C8	118.1 (3)	C15—C14—C19	119.7 (3)
C7—N1—Cu1	124.4 (2)	C15—C14—C13	117.6 (3)
C8—N1—Cu1	117.4 (2)	C19—C14—C13	122.6 (3)
C13—N2—C10	116.3 (3)	C16—C15—C14	120.5 (3)
C13—N2—Cu1	122.7 (2)	C16—C15—H15	119.7
C10—N2—Cu1	121.0 (2)	C14—C15—H15	119.7
O1—C1—C2	120.9 (3)	C15—C16—C17	120.9 (3)
O1—C1—C6	121.2 (3)	C15—C16—Cl2	120.3 (3)
C2—C1—C6	117.9 (3)	C17—C16—Cl2	118.8 (3)
C3—C2—C1	120.9 (3)	C18—C17—C16	119.7 (3)
C3—C2—H2	119.6	C18—C17—H17	120.1
C1—C2—H2	119.6	C16—C17—H17	120.1
C2—C3—C4	120.7 (3)	C17—C18—C19	121.5 (3)
C2—C3—H3	119.7	C17—C18—H18	119.2
C4—C3—H3	119.7	C19—C18—H18	119.2
C5—C4—C3	120.5 (3)	O2—C19—C18	120.1 (3)
C5—C4—Cl1	120.9 (3)	O2—C19—C14	122.3 (3)
C3—C4—Cl1	118.7 (3)	C18—C19—C14	117.6 (3)
C4—C5—C6	120.4 (3)	O3—C20—O4	127.0 (3)
C4—C5—H5	119.8	O3—C20—C21	118.1 (3)
C6—C5—H5	119.8	O4—C20—C21	115.0 (3)
C5—C6—C1	119.5 (3)	C20—C21—H21A	109.5
C5—C6—C7	118.3 (3)	C20—C21—H21B	109.5
C1—C6—C7	122.1 (3)	H21A—C21—H21B	109.5
N1—C7—C6	127.5 (3)	C20—C21—H21C	109.5
N1—C7—H7	116.2	H21A—C21—H21C	109.5
C6—C7—H7	116.2	H21B—C21—H21C	109.5
			
O2—Cu1—O1—C1	−137.9 (3)	Cu1—O1—C1—C2	156.4 (2)
N1—Cu1—O1—C1	34.1 (3)	Cu2—O1—C1—C2	3.4 (5)
N2—Cu1—O1—C1	−62.7 (4)	Cu1—O1—C1—C6	−25.2 (4)
O3—Cu1—O1—C1	131.8 (2)	Cu2—O1—C1—C6	−178.2 (2)
O2—Cu1—O1—Cu2	21.70 (10)	O1—C1—C2—C3	−178.6 (3)
N1—Cu1—O1—Cu2	−166.30 (11)	C6—C1—C2—C3	3.0 (5)
N2—Cu1—O1—Cu2	96.9 (3)	C1—C2—C3—C4	−0.9 (6)
O3—Cu1—O1—Cu2	−68.66 (10)	C2—C3—C4—C5	−2.3 (6)
O4^i^—Cu2—O1—C1	50.4 (3)	C2—C3—C4—Cl1	179.1 (3)
O4—Cu2—O1—C1	−129.6 (3)	C3—C4—C5—C6	3.3 (5)
O2^i^—Cu2—O1—C1	−40.8 (3)	Cl1—C4—C5—C6	−178.2 (3)
O2—Cu2—O1—C1	139.2 (3)	C4—C5—C6—C1	−1.1 (5)
O4^i^—Cu2—O1—Cu1	−107.81 (10)	C4—C5—C6—C7	−177.8 (3)
O4—Cu2—O1—Cu1	72.19 (10)	O1—C1—C6—C5	179.6 (3)
O2^i^—Cu2—O1—Cu1	161.04 (9)	C2—C1—C6—C5	−2.0 (5)
O2—Cu2—O1—Cu1	−18.96 (9)	O1—C1—C6—C7	−3.8 (5)
N1—Cu1—O2—C19	85.3 (6)	C2—C1—C6—C7	174.6 (3)
O1—Cu1—O2—C19	133.6 (3)	C8—N1—C7—C6	−176.5 (3)
N2—Cu1—O2—C19	−28.2 (3)	Cu1—N1—C7—C6	6.9 (5)
O3—Cu1—O2—C19	−135.0 (3)	C5—C6—C7—N1	−169.5 (3)
N1—Cu1—O2—Cu2	−67.7 (5)	C1—C6—C7—N1	13.9 (5)
O1—Cu1—O2—Cu2	−19.43 (9)	C7—N1—C8—C9	−113.3 (4)
N2—Cu1—O2—Cu2	178.80 (10)	Cu1—N1—C8—C9	63.6 (4)
O3—Cu1—O2—Cu2	71.96 (9)	N1—C8—C9—C11	54.7 (4)
O4^i^—Cu2—O2—C19	−41.8 (3)	N1—C8—C9—C10	−69.0 (4)
O4—Cu2—O2—C19	138.2 (3)	N1—C8—C9—C12	174.9 (3)
O1—Cu2—O2—C19	−132.1 (3)	C13—N2—C10—C9	134.9 (3)
O1^i^—Cu2—O2—C19	47.9 (3)	Cu1—N2—C10—C9	−46.4 (4)
O4^i^—Cu2—O2—Cu1	109.72 (10)	C11—C9—C10—N2	−64.1 (4)
O4—Cu2—O2—Cu1	−70.28 (10)	C8—C9—C10—N2	59.5 (4)
O1—Cu2—O2—Cu1	19.42 (9)	C12—C9—C10—N2	175.6 (3)
O1^i^—Cu2—O2—Cu1	−160.58 (9)	C10—N2—C13—C14	171.2 (3)
O2—Cu1—O3—C20	−48.2 (2)	Cu1—N2—C13—C14	−7.4 (5)
N1—Cu1—O3—C20	124.9 (2)	N2—C13—C14—C15	174.8 (3)
O1—Cu1—O3—C20	35.9 (2)	N2—C13—C14—C19	−9.1 (6)
N2—Cu1—O3—C20	−139.3 (2)	C19—C14—C15—C16	1.2 (5)
O1—Cu2—O4—C20	−44.3 (3)	C13—C14—C15—C16	177.4 (3)
O1^i^—Cu2—O4—C20	135.7 (3)	C14—C15—C16—C17	−1.4 (6)
O2^i^—Cu2—O4—C20	−149.7 (3)	C14—C15—C16—Cl2	177.8 (3)
O2—Cu2—O4—C20	30.3 (3)	C15—C16—C17—C18	1.4 (6)
O2—Cu1—N1—C7	23.8 (7)	Cl2—C16—C17—C18	−177.9 (3)
O1—Cu1—N1—C7	−24.2 (3)	C16—C17—C18—C19	−1.1 (5)
N2—Cu1—N1—C7	137.0 (3)	Cu1—O2—C19—C18	−159.7 (2)
O3—Cu1—N1—C7	−115.5 (3)	Cu2—O2—C19—C18	−16.4 (4)
O2—Cu1—N1—C8	−152.9 (5)	Cu1—O2—C19—C14	20.9 (4)
O1—Cu1—N1—C8	159.1 (2)	Cu2—O2—C19—C14	164.2 (2)
N2—Cu1—N1—C8	−39.6 (2)	C17—C18—C19—O2	−178.6 (3)
O3—Cu1—N1—C8	67.8 (2)	C17—C18—C19—C14	0.9 (5)
O2—Cu1—N2—C13	20.7 (3)	C15—C14—C19—O2	178.5 (3)
N1—Cu1—N2—C13	−149.5 (3)	C13—C14—C19—O2	2.5 (5)
O1—Cu1—N2—C13	−53.4 (5)	C15—C14—C19—C18	−0.9 (5)
O3—Cu1—N2—C13	111.5 (3)	C13—C14—C19—C18	−176.9 (3)
O2—Cu1—N2—C10	−157.9 (2)	Cu1—O3—C20—O4	0.0 (5)
N1—Cu1—N2—C10	31.9 (3)	Cu1—O3—C20—C21	179.9 (2)
O1—Cu1—N2—C10	128.0 (3)	Cu2—O4—C20—O3	6.2 (5)
O3—Cu1—N2—C10	−67.1 (3)	Cu2—O4—C20—C21	−173.7 (2)

Symmetry code: (i) −*x*+1, −*y*, −*z*+1.

### Hydrogen-bond geometry (Å, º)

**Table:**

*D*—H···*A*	*D*—H	H···*A*	*D*···*A*	*D*—H···*A*
C2—H2···O4^i^	0.93	2.58	3.115 (4)	117
C15—H15···O3^ii^	0.93	2.59	3.289 (4)	133

Symmetry codes: (i) −*x*+1, −*y*, −*z*+1; (ii) −*x*+3/2, *y*−1/2, *z*.
